# Supplemental oxygen does not improve growth but can enhance reproductive capacity of fish

**DOI:** 10.1098/rspb.2023.1779

**Published:** 2023-11-01

**Authors:** Michael R. Skeeles, Hanna Scheuffele, Timothy D. Clark

**Affiliations:** School of Life and Environmental Sciences, Deakin University, Geelong, Victoria 3216, Australia

**Keywords:** life history, climate change, gill surface area, gill-oxygen limitation theory (GOLT), hyperoxia, metabolic scaling‌

## Abstract

Fish tend to grow faster as the climate warms but attain a smaller adult body size following an earlier age at sexual maturation. Despite the apparent ubiquity of this phenomenon, termed the temperature-size rule (TSR), heated scientific debates have revealed a poor understanding of the underlying mechanisms. At the centre of these debates are prominent but marginally tested hypotheses which implicate some form of ‘oxygen limitation’ as the proximate cause. Here, we test the role of oxygen limitation in the TSR by rearing juvenile *Galaxias maculatus* for a full year in current-day (15°C) and forecasted (20°C) summer temperatures while providing half of each temperature group with supplemental oxygen (hyperoxia). True to the TSR, fish in the warm treatments grew faster and reached sexual maturation earlier than their cooler conspecifics. Yet, despite supplemental oxygen significantly increasing maximum oxygen uptake rate, our findings contradict leading hypotheses by showing that the average size at sexual maturation and the adult body size did not differ between normoxia and hyperoxia groups. We did, however, discover that hyperoxia extended the reproductive window, independent of fish size and temperature. We conclude that the intense resource investment in reproduction could expose a bottleneck where oxygen becomes a limiting factor.

## 1. Introduction

A decrease in the adult size of water-breathing ectotherms has emerged as a widespread response to climate warming [1,2]. Known as the temperature-size rule (TSR), ectotherms like fish tend to grow faster with warming but reach a smaller adult body size following an earlier onset of reproductive maturation [3]. Yet, despite the integral role of animal body size in ecosystem functioning [4], trophic interactions [5], fisheries and aquaculture productivity [6], and rural food security [7], the physiological mechanisms underlying the TSR remain speculative and hotly debated [8,9]. Unearthing the mechanisms underlying the TSR will help to refine conservation efforts and strengthen the forecasting power of statistical models in fisheries and aquaculture.

To date, some form of ‘oxygen limitation’ has been proposed as the key mechanism underlying the TSR in fish. For example, the prominent gill-oxygen limitation (GOL) hypothesis [10] implies that, at a certain size, the two-dimensional surface area of the gills ceases to supply sufficient oxygen for the growth demands of the three-dimensional body. Therefore, as warming increases maintenance oxygen requirements [11], the mismatch between oxygen supply and demand for growth transpires at a smaller body size. The GOL hypothesis also posits that, during ontogeny, sexual maturation is induced at the body size when the mismatch between oxygen supply and demand starts to occur (i.e. systemic hypoxia-induced maturation) [12]. Oxygen limitation as a driver of maturation implies there is a temperature-dependent response of tissues and the endocrine system to oxygen supply, which forms the premise of a somewhat related hypothesis – ‘maintain aerobic scope and regulate oxygen supply’ (MASROS) [13]. Aerobic scope is the difference between maintenance metabolism (≈ oxygen demand at rest) and maximum oxygen uptake (≈ oxygen supply capacity of the gills). Given that warming can reduce the aerobic capacity of larger individuals [11], MASROS suggests a size reduction in warmer temperatures allows fish to maintain their aerobic scope as a safety margin to prevent oxygen limitation [13]. If there is indeed some form of oxygen limitation throughout the life history of aquatic ectotherms, this is likely to be compounded by the continued decline in oxygen levels of the planet's aquatic ecosystems [14].

Despite significant global attention given to hypotheses about oxygen limitation, there remains little consensus as to whether oxygen limitation can provide a ‘universal’ explanation for the TSR [8,15,16]. Another possibility is that the TSR is a size-dependent shift in life-history strategy to maximize lifetime reproductive success—the primary evolutionary goal of all organisms. Given that mortality generally increases in warmer environments [17,18], diverting energy towards an earlier onset of sexual maturation with warming—albeit at a smaller size—may increase the probability of reproducing [19]. On the other hand, the lower mortality in cooler settings [18] could give individuals the opportunity to mature later, but at a larger size, thereby benefiting from an increased lifetime reproductive output [20]. Rather than any immediate temperature-induced physiological constraint, these considerations imply that the TSR may be the outcome of adaptive energy allocation and life-history optimization [21,22]. Oxygen limitation may still be implicated, but as an ultimate rather than proximate mechanism. This has been described as the ‘ghost of oxygen-limitation past’ [23], which suggests TSR responses have evolved to ensure sufficient oxygen provisioning under warming because selection has acted against those growth-reproductive genotypes that experienced imbalances between oxygen supply and demand during previous episodic hypoxia and warming events [24].

Although existing evidence provides some support for each of the constraint-based and life-history optimization-based explanations, none of these mechanisms appear to be broadly applicable [9]. A lack of consensus generally stems from the challenges involved in quantifying lifetime growth and reproduction in fishes under manipulated environmental conditions [25]. Hence, our current understanding largely relies on TSR-like responses of small fishes that mature quickly [26], acute studies on larger fish [27] or correlations from the wild [2]. It is widely accepted that long-term growth studies with manipulated temperature and oxygen levels are needed on species beyond the usual model organisms to address whether growth is oxygen limited in warmer settings [24].

Here, we assessed the growth and maturation responses of a fish to warming and varying levels of oxygen availability. We reared wild-caught juvenile *Galaxias maculatus* for a year at a current-day summer mean temperature of 15°C and a forecasted summer mean temperature of 20°C, and provided half of each temperature group with supplementary oxygen (150% air saturation). We quantified metabolic traits of fish from each treatment, measured their growth every 2 months, and developed a non-destructive method to monitor temporal dynamics of sexual maturation and reproductive investment. In line with the TSR, we expected to see an earlier onset of sexual maturation at a smaller size for fish at 20°C compared with 15°C. Moreover, based on oxygen limitation hypotheses, we expected individuals from the 20°C hyperoxia treatment to grow larger and exhibit better reproductive potential than their normoxic counterparts, as they had access to supplementary oxygen that would improve their oxygen uptake capacity [28,29] and alleviate any temperature-induced constraints.

## 2. Material and methods

### (a) Model species

The common galaxiid *Galaxias maculatus* is a widely distributed salmoniform fish in the Southern Hemisphere with a key role in New Zealand and South American fisheries as well as marine, estuarine and freshwater ecosystems [30]. Larval *G. maculatus* in southeastern Australia spend approximately 5 months at sea before migrating into estuaries as unpigmented juveniles (commonly known as whitebait) in October/November [31]. In estuaries, and upstream, they develop into adults and usually reach reproductive maturity, at a range of sizes, by July in the subsequent year [31]. Post-spawning mortality is generally high [32] but individuals that survive can live and reproduce for up to 3 or 4 years [33,34], attaining a maximum size of 190 mm total length (TL) [35]. The species is considered to be an adaptable opportunist, with the ability to adjust life-history strategies to the requirements of the prevailing environment [33].

### (b) Animal capture and acclimation

Full methods are provided in the supplementary information (electronic supplementary material, appendix SI) but are summarized here. In early November 2020, juvenile *G. maculatus* were caught in the Cumberland River mouth, Lorne, Victoria, Australia, as they returned from their larval phase at sea (species details in electronic supplementary material, appendix SI). Across 2 days, approximately 1000 fish were caught using box traps baited with cat food (trap dimensions (L × W × H): 480 × 250 × 250 mm; water temperature: 14.5–16°C; salinity: 3–10 ppt). Fish were transported by road to Deakin University's Queenscliff Marine Science Centre and were left to habituate to captivity at 16.5°C in two 125 l aerated tanks continuously supplied with clean water (flow rate: 80 l h^−1^; salinity: approx. 7 ppt). During this time, fish were weaned onto a commercial diet (Otohime; BMAQUA, Frederickton, New South Wales, Australia).

On 13 and 14 January 2021, 880 fish were evenly distributed across four racks, each housing 10 25 l rectangular replicate tanks. Within each rack, tanks drained into a 200 l sump equipped with a protein skimmer, a mechanical and a biological filter. Water then passed through a UV sterilizer in transit back to the tanks from a supply pump in the sump. This extensive filtration of water during each cycle ensured that each tank on the rack could be treated as an independent replicate. A minimum of 50% of total water volume in each rack system was replaced each week. Each replicate tank was stocked with 22 individuals (means ± s.d.; TL = 54 ± 4 mm, mass = 1.17 ± 0.41 g) such that biomass was consistent across tanks. At the time of distribution, water parameters were equivalent to the habituation conditions (i.e. 16.5°C and approx. 7 ppt). After a day, temperature was altered in each rack to achieve 15°C in two racks and 20°C in the other two. Temperature change was no greater than 0.25°C h^−1^ and fish were left to adjust to their new thermal conditions for a week before hyperoxia was induced (within an hour) in two of the racks using regulated bubbling from oxygen gas bottles. Thus, the four treatments were: cool normoxia (15°C, 100% air saturation (termed dissolved oxygen (DO) herein)), cool hyperoxia (15°C, 150% DO), warm normoxia (20°C, 100% DO) and warm hyperoxia (20°C, 150% DO; electronic supplementary material, appendix SI*,* electronic supplementary material, figure S1). Fish were reared in these treatments for 12 months (the duration of the 2021 calendar year; electronic supplementary material, figure S1), although half of the tanks were diverted to a parallel study from September onwards (details in electronic supplementary material).

The two temperatures were selected to approximate the Cumberland River's historical summer mean (15°C), and a current-day summer extreme temperature that is expected to become more common throughout this century (20°C; see electronic supplementary material, figure S2*a*). Hyperoxia levels were chosen based on the upper range recorded in the Cumberland River (electronic supplementary material, figure S2*b*), when photosynthetic activity was high. Temperature and oxygen conditions were monitored daily with a YSI Pro2030 handheld meter (YSI Incorporated, Yellow Springs, Ohio, USA) that was routinely cross-referenced with other YSI sensors. Fish were fed to satiation 5 days a week and starved 48 h prior to any experimentation. The photoperiod was set by LED lighting and was adjusted through time to track the conditions at the capture location (electronic supplementary material, figure S1), including a simulated sunrise and sunset by progressively lighting and dimming for 1 h in the mornings and evenings, respectively, to ensure fish were not startled by abrupt lighting changes.

### (c) Growth and maturation

**Figure 1.**
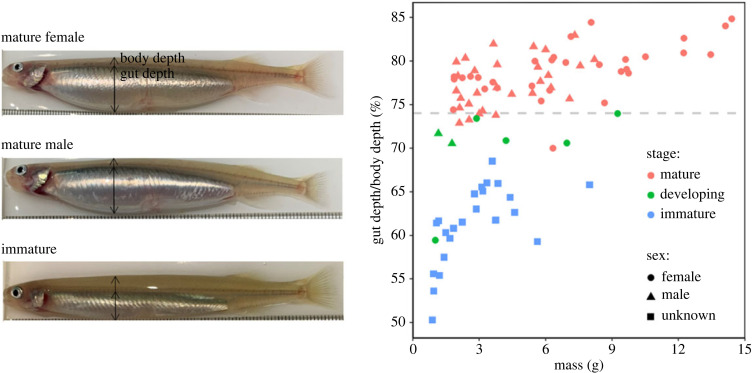
Gut : body depth ratio of 91 *Galaxias maculatus* that were dissected to macroscopically confirm their maturation stage (immature, developing or mature). These were plotted per sex and across body mass to illustrate, regardless of these parameters, a threshold (74%, dashed line) between mature and immature/developing fish. Photographs are examples of a mature female, mature male and an immature fish, and the positions at which the gut depth and body depth measurements were taken. Note the eggs within the ovaries of the mature female and the glistening white testes of the mature male, which allowed sex determination when fish were mature.

Length and mass of all fish were recorded every 2 months from January 2021 following sedation with Aqui-S (electronic supplementary material, figure S1). When recording length and mass in May, July, September and November, we photographed a random subset of fish while on the measuring trough (electronic supplementary material, figure S1). Photos only began for each treatment when there were distinct signs of mature individuals. Photos were linked to an individual's length and mass through the camera's integrated time-stamp. There were distinct signs of gonadal development in *G. maculatus* through bulging of the gut cavity (figure 1), which allowed us to develop a high-throughput method to confirm whether sedated individuals were mature or not.

First, we calibrated a relationship between morphometrics and maturation level by sacrificing a subset of fish. Over May, June and July we photographed and dissected 91 fish with varying gut sizes, across all masses and treatments, to evaluate whether there was a relationship between gross body morphology and an individual's maturation level. Fish were photographed, euthanized in an ice-slurry, their gonads weighed, and their maturation condition staged macroscopically (following the criteria of Stevens *et al*. [34]). From the photographs, we calculated gut cavity length, depth and area, and body length and depth for each fish using ImageJ [36], and linked these with the individual's maturation level. Regardless of sex, mass or treatment we found a distinct relationship between the gut depth to body depth ratio (converted to %) of an individual and their maturation level (figure 1). Fish with a ratio of 74% and above were all mature (figure 1). To statistically confirm this, we fitted a logistic regression model between gut : body depth and maturation probability, which indicated that a fish with a ratio of ≥74% had a ≥98% (95% CI, 93–100%) probability of being mature.

We subsequently measured the gut : body depth ratio of each photographed individual during our bimonthly weighing periods and, based on the threshold value of 74%, assigned them a maturation level of either mature (ratio ≥74%) or immature (ratio <74%). This enabled us to monitor body size at reproductive maturation and the length of the reproductive period in each treatment. If fish were mature, their sex was recorded through externally visible eggs in the ovaries or a glistening white belly indicating testes (figure 1).

### (d) Metabolism

To understand the effects of our experimental treatments on oxygen requirements and metabolic capacities, we conducted respirometry trials in June (electronic supplementary material, figure S1). Through measurements of oxygen uptake rates $\left({\dot{M}}_{O_{2}}\right)$, we quantified the maximum oxygen uptake capacity (≈ maximum aerobic metabolic rate (MMR)) and oxygen requirements for maintenance (≈ standard metabolic rate (SMR)) of a subset of fish (*n* = ∼14) for each treatment at their respective conditions and across the full size range (0.87–15.12 g). In short, individuals were removed from their tank, exercised to exhaustion using a 2 min chase protocol under their acclimated conditions, weighed, and immediately placed in a sealed respirometer to capture their MMR. Subsequently, respirometers were intermittently flushed and sealed overnight for approximately 18 h to record SMR. All details are given in the electronic supplementary material.

### (e) Statistical analyses

#### (i) Oxygen uptake rates

To compare the maximum oxygen uptake capacity and oxygen maintenance requirements between treatments (hyperoxia versus normoxia) and across body mass for each temperature group, we used linear models. The measured trait (log-transformed) was the response variable and mass (always log-transformed), treatment (hyperoxia) and their full interaction (hyperoxia × mass) were the predictor variables. We then tested if maintenance requirements (log-transformed) differed between temperatures (cool versus warm) for each oxygen group by using temperature, mass and their full interaction as fixed effects. Note, we visually present these metabolic data in a mass-specific format to align our results with the typical curves used in the GOL hypothesis to demonstrate a decrease in relative oxygen supply with body mass.

#### (ii) The explanatory power of temperature and/or oxygen effects on growth: model selection

All growth analyses were conducted on tanks monitored throughout the entire year and not those excluded after September. To test whether growth was modulated by temperature and/or oxygen, we separately modelled (using maximum-likelihood estimation) length and mass (both log-transformed) as a function of the full interaction between temperature (15°C versus 20°C), oxygen (normoxia versus hyperoixa) and time (days, third order polynomial) with replicate tank nested in treatment group (cool-normoxia, cool-hyperoxia, warm-normoxia and warm-hyperoxia) as a random intercept to account for repeated measures. Subsequently, either the interactive or full oxygen/ temperature terms were dropped from the complex model to assess whether this yielded better explanatory power (lower Akaike information criterion (AICc)), thereby illuminating the importance of each factor for growth. The highest explanatory models were considered those with the lowest AICc and models with ΔAICc of <2 were considered statistically similar.

#### (iii) Oxygen effects on growth and reproduction at independent temperatures

Given growth trajectories differed under cool and warm conditions (see results), and that the focus of the study was to address whether oxygen can improve growth particularly under warm conditions, we split the temperature groups for further investigation.

*Estimated marginal means and pairwise analyses.* For each temperature group, linear mixed effects models were used to compare the length and mass of fish between oxygen treatments (normoxia versus hyperoxia) and time points (measurement period, 1–7 as a categorical variable). This entailed fitting log-transformed length/ mass as a response to the interaction between time and oxygen, with replicate tank as a random intercept to account for repeated measures. We then used a type III analysis of variance test to assess the significance of each predictor variable (alpha = 0.05). Subsequently, to extract and compare estimated marginal means of these models (i.e. *post hoc* test), we used a Bonferroni multiple comparison test (emmeans package [37]) and a compact letter display to visually show any differences.

Maturation analyses were based on all 10 tanks from each treatment up until September, and the remaining five tanks per treatment dedicated to this study thereafter. For the maturation of fish, linear mixed models were used to assess the interactive effects of oxygen (normoxia versus hyperoxia) and time (measurement period, categorical) on maturation level (gut width: body depth % ratio) at each independent temperature, with tank as a random intercept. Estimated marginal means were evaluated as per the method above. The size of fish at sexual maturation was then compared using a mixed model with mass-at-maturation as a function of the full interaction between sex and oxygen, with replicate tank as a random intercept. Where possible, sex ratios were compared between treatments using a chi-squared test.

All statistical analyses were conducted in R [38] and linear mixed effects models were fit with the Lmer package [39]. Linear model assumptions of residual normality and homogeneity of variance were checked using a series of residual plots.

*Growth/maturation models with time as a continuous variable.* In addition to estimated marginal mean comparisons, we ran a series of mixed models where oxygen could interact with time as a continuous variable (days, 0–330) to generate smoothed growth/maturation curves. For ease of interpretation, we do not refer to these models in the results but rather use their outputs for graphical representation of the growth/maturation trends. All statistical methods and outputs pertaining to these models can be found in the electronic supplementary material. Note, the effect of oxygen in these models did not differ from the main models where time was a categorical factor.

## 3. Results

### (a) Oxygen uptake capacity and maintenance requirements

**Figure 2.**
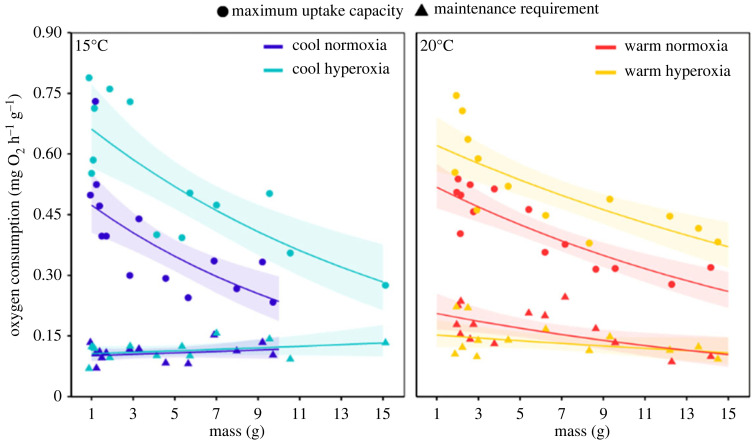
Mass-specific maximum oxygen uptake capacity (maximum aerobic metabolic rate; circles) and oxygen requirements for maintenance metabolism (standard metabolic rate; triangles) across body mass for *Galaxias maculatus* reared in normoxia (100% DO) and hyperoxia (150% DO) treatments at cool (15°C) and warm (20°C) temperatures. Lines represent best fit relationships with shaded 95% CI. Note, statistical analyses were conducted on raw metabolic data but, to keep the plots consistent with those typically presented by proponents of the GOL hypothesis, we present the data here in a mass-specific format.

At both cool and warm temperatures, hyperoxia-reared fish across all tested sizes had a significantly elevated maximum ${\dot{M}}_{O_{2}}$ compared with normoxia-reared counterparts (figure 2 and electronic supplementary material, table S1). Oxygen requirements for maintenance metabolism did not differ between normoxia- and hyperoxia-reared fish (figure 2 and electronic supplementary material, table S1). Within normoxia treatments, maintenance costs were significantly greater at 20°C than at 15°C, although this temperature-related difference in maintenance metabolism was not detected within hyperoxia treatments (figure 2 and electronic supplementary material, table S1). Similarly, temperature increased maximum uptake capacity in normoxia but no differences were evident in hyperoxia (figure 2 and electronic supplementary material, table S1).

### (b) Growth

**Figure 3.**
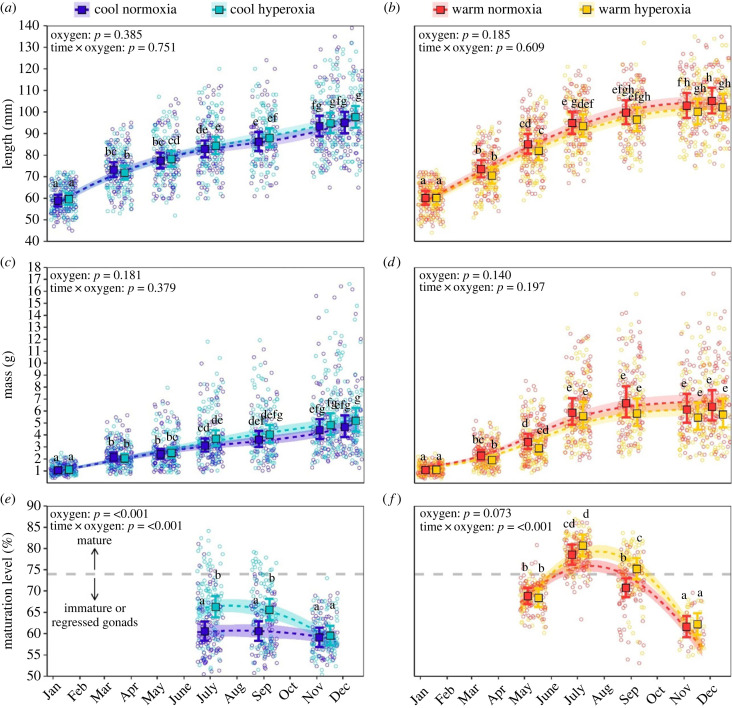
Growth in length (*a,b*), mass (*c,d*) and maturation level (*e,f*) over the course of a year for juvenile *Galaxias maculatus* reared in 15°C normoxia, 15°C hyperoxia, 20°C normoxia and 20°C hyperoxia treatments, each comprising five replicate tanks (*n* ≈ 110 fish per treatment). Circles indicate individual measurements and squares denote the overall estimated marginal means (±95% CI) for each measurement period calculated from respective mixed effects models. The significance of oxygen and the interaction between oxygen and time on each trait are indicated in the top left. Compact letter displays are separate for each panel and means not sharing letters are significantly different from one another (*α* = 0.05). For *e* and *f*, the maturation level is a per cent ratio of an individual's gut depth to overall body depth. Here, the grey dashed line represents the calculated threshold (74%) for which fish are either mature (≥74%) or immature/gonads have regressed (<74%). Coloured dashed lines are derived from mixed model predictions (shaded 95% CIs) when time was a continuous predictor (see supplementary material).

Temperature had a strong influence on the growth (length and mass) of fish over the experiment, evidenced by the temperature × time interaction having the highest explanatory power in the model selection protocol (table 1). If growth was oxygen limited at warm temperatures, we would expect the time × temperature × oxygen model to have the highest explanatory power, yet including oxygen as a predictor made no improvements to both models' explanatory power (table 1). The absence of an oxygen effect on growth was further evident in the outputs of temperature-specific growth models, which revealed no significant oxygen effect, or interaction between oxygen and time, on length and mass (figure 3*a–d*) indicating growth was not oxygen limited in normoxia.

**Table 1.** Model selection based on corrected Akaike information criterion (AICc) for relevant linear mixed models addressing the most important explanatory variables of the growth of juvenile *Galaxias maculatus* in length and mass (both log-transformed) over time (days, third-order polynomial). K is the number of parameters, ΔAICc is the difference between AICc of the respective model and the best model, w_i_AICc is the probability that the respective model is the best model and LL is log likelihood. Multiplication signs denote a full interaction with oxygen whereas the addition symbol signifies just the overall effect of oxygen.

model components	*K*	AICc	ΔAICc	w_i_AICc	LL
**length**					
temperature × time	11	−7959.51	0.00	0.62	3990.81
temperature × time + oxygen	12	−7958.22	1.29	0.33	3991.17
temperature × time × oxygen	19	−7954.40	5.11	0.05	3996.34
time	7	−7881.59	77.93	0.00	3947.81
time × oxygen	11	−7878.54	80.98	0.00	3950.32
**mass**					
temperature × time	11	−977.22	0.00	0.42	499.66
temperature × time × oxygen	12	−977.20	0.02	0.41	507.74
temperature × time + oxygen	19	−975.38	1.84	0.17	499.75
time	7	−872.47	104.75	0.00	443.26
time × oxygen	11	−870.35	106.87	0.00	446.22

Corresponding to the temperature-size rule, growth was slow and steady in normoxia- and hyperoxia-reared fish at 15°C with gradual increases in length and mass occurring throughout the year (figure 3*a,c*). At 20°C, however, growth was initially rapid in both oxygen treatments with significant increases in length and mass detectable every 2 months, yet growth started to wane around July and did not recover after September (figure 3*b,d*). These growth trends corresponded with periods of maturation, as detailed below.

### (c) Maturation

The rate of reproductive maturation differed between temperature treatments. The first signs of individuals reaching maturity were earlier for warm treatments (May) compared with cool treatments (July) (figure 3*e,f*). Throughout the year, the average maturation level for both 15°C treatments remained below 74%, the value indicative of being mature (figure 3*e,f*). Despite significant increases in the maturation level of some 15°C fish (particularly those in hyperoxia) in July and September, the majority of fish at 15°C never reached sexual maturation within the year (figure 3*e*). At 20°C, the average fish was still immature in May but by July almost all individuals were reproductively mature (figure 3*f*). For normoxia- and hyperoxia-reared fish at 20°C, 86% and 94% of fish were mature in July, respectively, whereas for equivalent oxygen treatments at 15°C, only 5% and 19% were mature.

**Figure 4.**
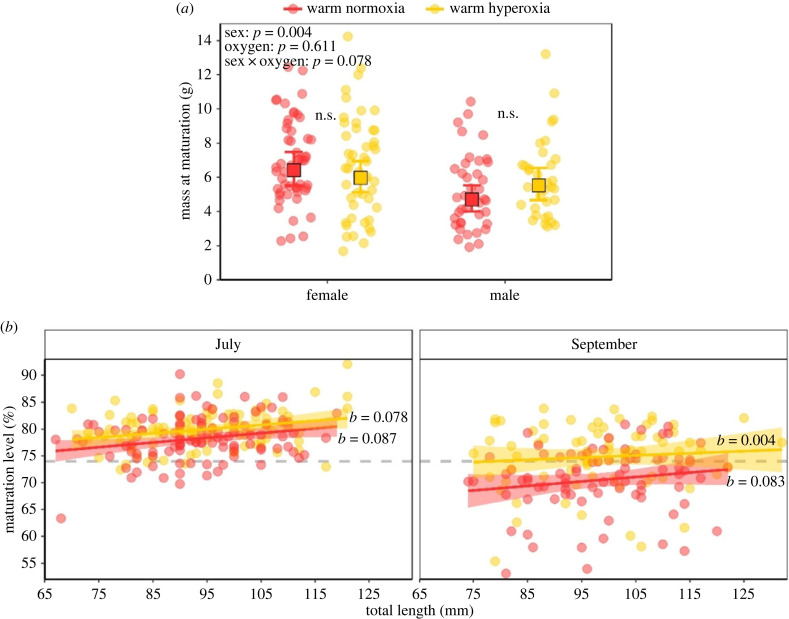
(*a*) The mass at reproductive maturation for female and male *Galaxias maculatus* from 20°C normoxia and 20°C hyperoxia treatments. Circles represent raw data and squares are estimated marginal means (±95% CI) from the mixed model (significance of factors from the same model presented in the top left). *NS* stands for not significantly different. (*b*) Maturation level of fish from 20°C normoxia and 20°C hyperoxia treatments in the peak reproductive months of July and September. Raw values (circle symbols) are plotted across body length with respective best-fit linear relationships (*y* = a + bx) and shaded standard error. This is to illustrate that the temporal extension of the reproductive window in hyperoxia was not size dependent. Slopes of relationships (*b*) are presented and the dashed grey line represents the calculated threshold of the gut : body depth ratio (74%) for which fish are either mature (≥74%) or immature/gonads have regressed (<74%).

At 20°C, the overall sex ratio of fish did not differ between oxygen treatments (chi-squared = 1.55, d.f = 1, *p* = 0.21). Females were larger than males at maturation and oxygen availability had no effect on the size at which either sex matured at 20°C (figure 4*a*). Following the reproductive peak of 20°C fish in July, the average maturation level significantly dropped in September (figure 3*f*), indicating either the release or regression of gonads. Here, however, additional oxygen seemed to extend the reproductive window, as 64% of fish still carried ripe gonads compared with 31% of their normoxia-reared counterparts (figure 3*f*). This oxygen-linked difference in the reproductive window was also evident in the 15°C fish (figure 3*e*). As such, the average maturation level in September significantly differed between oxygen treatments for both temperatures (figure 3*e,f*). Notably, at 20°C, the hyperoxia-induced extension of the reproductive window was independent of fish size (figure 4*b*). The reproductive season had finished by November, as no fish in any of the treatments were carrying ripe gonads (figure 3*e,f*). This reproductive window (July to September) parallels the natural window for *G. maculatus* in the Cumberland River [31].

## 4. Discussion

To address a heated debate about the proximate role of oxygen in the TSR phenomenon, we quantified the growth and reproductive trajectories of wild-caught *G. maculatus* for the first year of their post-larval life in response to warming and supplementary oxygen (hyperoxia). We found thermal trends consistent with the TSR [25,26], in that individuals in warm conditions grew faster and reached sexual maturation earlier, and also plateaued in length and mass. By contrast, cool-reared conspecifics maintained a roughly linear growth trajectory and the majority appeared on track to mature in the following year's reproductive window. Hyperoxia increased oxygen supply capacity (as shown previously [29]) but that improvement did not translate into a larger size at sexual maturation or adult body size with warming, providing evidence against multiple theories that implicate a direct role of oxygen limitation in driving the TSR. However, we found evidence that the reproductive window can be extended in hyperoxia, raising the possibility that the reproductive capacity of fish may be oxygen limited under normoxic conditions. Given the climate warming-induced changes to the energy budgets of fish, our findings suggest that reproduction could represent a tightening bottleneck in the life history of fishes in a changing climate.

### (a) Oxygen-limited growth?

Current explanations of the TSR have centred on some form of physiological constraint resulting in proximate oxygen limitation. Hyperoxia increased the maximum oxygen uptake capacity of exercising *G. maculatus* by approximately 35%, indicating that individuals had the capacity to transport and use the additional oxygen in their holding tanks to accommodate daily activities including somatic growth. Thus, despite enhanced access to oxygen and the physiological capacity to use it, TSR trends still prevailed and the growth and maturation size of warm-hyperoxia fish paralleled those in warm-normoxia. Similarly, hyperoxia did not improve adult growth of *G. maculatus* in Skeeles and Clark [40], nor did it circumvent TSR trends of several aquatic insects in Funk *et al*. [41]. The effects of hyperoxia on the growth performance of fishes has recently been reviewed by McArley *et al*. [28]. Of 30 studies, only 7 reported improved growth under hyperoxia, and 3 of these may be somewhat misleading because they made comparisons with a control group under mildly hypoxic conditions (76–80% DO). However, the review did not examine interactive effects with temperature, which leaves some unanswered questions given that any hyperoxia-induced growth improvements may be expected to manifest with warming. Nonetheless, 20 of the 30 available studies reported negligible effects of hyperoxia on growth, corroborating our findings and suggesting that growth in normoxia is not oxygen limited.

Our use of maximum oxygen uptake capacity (MMR) as a proxy for gill surface area (GSA) provides a test of the GOL hypothesis – the most prominent yet hotly debated explanation of the TSR in fish [8,12]. If GSA universally limits the growth of fish due to oxygen supply limitations, then the hyperoxia-induced increase in MMR observed in *G. maculatus* would have propelled a larger size at maturation and adult body size, but it did not. Our results support a recent analysis showing that the allometric scaling relationships for GSA and metabolism do not suggest a progressive impairment of gill-oxygen supply as fish grow [42].

### (b) Oxygen-limited reproduction?

Although supplementary oxygen did not influence growth or size at maturation, it did extend the reproductive window for both warm- and cool-reared fish (figure 3*e,f*). This is a novel finding that suggests the reproductive period exposes a vulnerable link in the oxygen transport systems of fish. Indeed, the reproductive process may be the most physiologically challenging period in the life history of many fish [43], given that individuals have to bear the additional burden of metabolically active gonads [44] for weeks or months on top of typical daily demands. While some research attention has been given to the detrimental effects of hypoxia on reproductive processes [45,46], the potential benefits of hyperoxia on reproductive capacity have received scant attention, although it has been reported that hyperoxia may increase the follicle diameter of mature female sterlet *Acipenser ruthenus* [47]. Nevertheless, despite the potential for some level of oxygen limitation in the reproductive process, we show that the temporal extension of the reproductive window in *G. maculatus* was size independent (see figure 4), contradicting the foundations of direct oxygen-limited explanations of the TSR. Our study represents a step towards better understanding the connection between oxygen availability and reproduction, which is suggested to be a critical life-history phase for genotypic selection and the potential evolution of TSR reaction norms over time (see Marshall and White [16] and ‘ghost of oxygen-limitation past’ in Verberk *et al*. [23]).

### (c) Conclusions and future directions

Almost three decades after the TSR was first described [3], the underlying mechanisms remain inadequately explained. While the most prominent explanations imply oxygen limitation as the proximate cause, we find that TSR trends persist in *G. maculatus* despite a year of early-life oxygen enrichment. These findings suggest other mechanisms are in action, and it may be that the TSR is not a result of a direct physiological limitation but instead is a consequence of temperature-mediated energy reallocation to optimize life history [21,40]. Whether this temperature-induced diversion of energy is mostly genetically or plastically modulated is a topical question [48] and perhaps monitoring growth while manipulating environmental triggers for reproduction (e.g. photoperiod) could disentangle the two regulatory mechanisms.

Much of the discussion around oxygen limitation in the TSR phenomenon draws on examples from studies using low oxygen (hypoxia) and extrapolating up to normoxic conditions. For example, Kolding *et al*. [49] found a decrease in the maturation and adult body size of Nile tilapia *Oreochromus niloticus* under hypoxic conditions, which other studies (e.g. Pauly [12]) commonly cite as evidence to support the GOL hypothesis. However, depriving fish of normal oxygen levels may trigger a range of physiological processes (e.g. gill remodelling [50]) that divert resources away from somatic growth, leading to a flawed understanding of the role of oxygen in the TSR under normoxic conditions. We echo the suggestion of Atkinson *et al*. [24] that mild levels of hyperoxia can better reveal whether ectotherm life in normoxia may be oxygen limited, especially given that supplementary oxygen can improve cardiorespiratory capacities of fish with no apparent cost to maintenance oxygen requirements [29] (figure 2).

In sum, this study provides robust evidence against theories implicating proximate oxygen limitation in driving the TSR. However, as far as we are aware, we reveal for the first time a size- and temperature-independent oxygen limitation during the reproductive period which acts to reduce the reproductive window in normoxia compared with hyperoxia. Such oxygen-limited reproduction could represent a tightening bottleneck for fishes as climate warming continues to alter their energy budgets.

## Data Availability

The data supporting these findings and the analyses code are publicly available on Figshare at https://doi.org/10.6084/m9.figshare.21677174 [51]. Supporting methods and results are provided in the electronic supplementary material [52].

## References

[1] Gardner JL, Peters A, Kearney MR, Joseph L, Heinsohn R. 2011 Declining body size: a third universal response to warming? Trends Ecol. Evol. **26**, 285-291. (10.1016/j.tree.2011.03.005) 21470708

[2] Audzijonyte A, Richards SA, Stuart-Smith RD, Pecl G, Edgar GJ, Barrett NS, Payne N, Blanchard JL. 2020 Fish body sizes change with temperature but not all species shrink with warming. Nat. Ecol. Evol. **4,** 1-6. (10.1038/s41559-020-1171-0) 32251381

[3] Atkinson D. 1994 Temperature and organism size: a biological law for ectotherms? Adv. Ecol. Res. **25**, 1-58. (10.1016/S0065-2504(08)60212-3)

[4] Sheridan JA, Bickford D. 2011 Shrinking body size as an ecological response to climate change. Nat. Clim. Change **1**, 401-406. (10.1038/nclimate1259)

[5] Audzijonyte A, Kuparinen A, Gorton R, Fulton EA. 2013 Ecological consequences of body size decline in harvested fish species: positive feedback loops in trophic interactions amplify human impact. Biol. Lett. **9**, 20121103. (10.1098/rsbl.2012.1103) 23365151 PMC3639762

[6] Barneche DR, Jahn M, Seebacher F. 2019 Warming increases the cost of growth in a model vertebrate. Funct. Ecol. **33**, 1256-1266. (10.1111/1365-2435.13348)

[7] Oke K et al. 2020 Recent declines in salmon body size impact ecosystems and fisheries. Nat. Commun. **11**, 1-13. (10.1038/s41467-019-13993-7) 32814776 PMC7438488

[8] Lefevre S, Mckenzie DJ, Nilsson GE. 2017 Models projecting the fate of fish populations under climate change need to be based on valid physiological mechanisms. Glob. Change Biol. **23**, 3449-3459. (10.1111/gcb.13652) 28168760

[9] Audzijonyte A, Barneche DR, Baudron AR, Belmaker J, Clark TD, Marshall CT, Morrongiello JR, Van Rijn I. 2019 Is oxygen limitation in warming waters a valid mechanism to explain decreased body sizes in aquatic ectotherms? Glob. Ecol. Biogeogr. **28**, 64-77. (10.1111/geb.12847)

[10] Pauly D. 1981 The relationships between gill surface area and growth performance in fish: a generalization of von Bertalanffy's theory of growth. Meeresforschung **28**, 251-282.

[11] Rubalcaba JG, Verberk WC, Hendriks AJ, Saris B, Woods HA. 2020 Oxygen limitation may affect the temperature and size dependence of metabolism in aquatic ectotherms. Proc. Natl Acad. Sci. USA **117**, 31 963-31 968. (10.1073/pnas.2003292117) PMC7749359 33257544

[12] Pauly D. 2021 The gill-oxygen limitation theory (GOLT) and its critics. Sci. Adv. **7**, eabc6050. (10.1126/sciadv.abc6050) 33523964 PMC7787657

[13] Atkinson D, Morley SA, Hughes RN. 2006 From cells to colonies: at what levels of body organization does the ‘temperature-size rule' apply? Evol. Dev. **8**, 202-214. (10.1111/j.1525-142X.2006.00090.x) 16509898

[14] Schmidtko S, Stramma L, Visbeck M. 2017 Decline in global oceanic oxygen content during the past five decades. Nature **542**, 335-339. (10.1038/nature21399) 28202958

[15] Clark TD, Sandblom E, Jutfelt F. 2013 Aerobic scope measurements of fishes in an era of climate change: respirometry, relevance and recommendations. J. Exp. Biol. **216**, 2771-2782. (10.1242/jeb.084251) 23842625

[16] Marshall DJ, White CR. 2019 Aquatic life history trajectories are shaped by selection, not oxygen limitation. Trends Ecol. Evol. **34**, 182-184. (10.1016/j.tree.2018.12.015) 30638913

[17] Pauly D. 1980 On the interrelationships between natural mortality, growth parameters, and mean environmental temperature in 175 fish stocks. ICES J. Mar. Sci. **39**, 175-192. (10.1093/icesjms/39.2.175)

[18] Álvarez-Noriega M, White C, Kozłowski J, Day T, Marshall D. 2023 Life history optimisation drives latitudinal gradients and responses to global change in marine fishes. PLoS Biol. **21**, e3002114. (10.1371/journal.pbio.3002114) 37228036 PMC10212075

[19] Cole LC. 1954 The population consequences of life history phenomena. Q Rev. Biol. **29**, 103-137. (10.1086/400074) 13177850

[20] Barneche DR, Robertson DR, White CR, Marshall DJ. 2018 Fish reproductive-energy output increases disproportionately with body size. Science **360**, 642-645. (10.1126/science.aao6868) 29748282

[21] Kozłowski J. 1992 Optimal allocation of resources to growth and reproduction: implications for age and size at maturity. Trends Ecol. Evol. **7**, 15-19. (10.1016/0169-5347(92)90192-E) 21235937

[22] Kozłowski J, Czarnołęski M, Dańko M. 2004 Can optimal resource allocation models explain why ectotherms grow larger in cold? Integr. Comp. Biol. **44**, 480-493. (10.1093/icb/44.6.480) 21676734

[23] Verberk WC, Atkinson D, Hoefnagel KN, Hirst AG, Horne CR, Siepel H. 2021 Shrinking body sizes in response to warming: explanations for the temperature–size rule with special emphasis on the role of oxygen. Biol. Rev. **96**, 247-268. (10.1111/brv.12653) 32959989 PMC7821163

[24] Atkinson D, Leighton G, Berenbrink M. 2022 Controversial roles of oxygen in organismal responses to climate warming. Biol. Bull. **243**, 207-219. (10.1086/722471) 36548977

[25] Wootton HF, Morrongiello JR, Schmitt T, Audzijonyte A. 2022 Smaller adult fish size in warmer water is not explained by elevated metabolism. Ecol. Lett. **25**, 1177-1188. (10.1111/ele.13989) 35266600 PMC9545254

[26] Loisel A, Isla A, Daufresne M. 2019 Variation of thermal plasticity in growth and reproduction patterns: importance of ancestral and developmental temperatures. J. Therm. Biol. **84**, 460-468. (10.1016/j.jtherbio.2019.07.029) 31466787

[27] Messmer V, Pratchett MS, Hoey AS, Tobin AJ, Coker DJ, Cooke SJ, Clark TD. 2017 Global warming may disproportionately affect larger adults in a predatory coral reef fish. Glob. Change Biol. **23**, 2230-2240. (10.1111/gcb.13552) 27809393

[28] Mcarley TJ, Sandblom E, Herbert NA. 2021 Fish and hyperoxia—from cardiorespiratory and biochemical adjustments to aquaculture and ecophysiology implications. Fish Fish. **22**, 324-355. (10.1111/faf.12522)

[29] Skeeles M, Scheuffele H, Clark T. 2022 Chronic experimental hyperoxia elevates aerobic scope: a valid method to test for physiological oxygen limitations in fish. J. Fish Biol. **101**, 1595-1600. (10.1111/jfb.15213) 36069991 PMC10087569

[30] Mitchell CP. 1989 Laboratory culture of *Galaxias maculatus* and potential applications. New Zealand J. Mar. Freshw. Res. **23**, 325-336. (10.1080/00288330.1989.9516369)

[31] Barbee NC, Hale R, Morrongiello J, Hicks A, Semmens D, Downes BJ, Swearer SE. 2011 Large-scale variation in life history traits of the widespread diadromous fish, *Galaxias maculatus*, reflects geographic differences in local environmental conditions. Mar. Freshw. Res. **62**, 790-800. (10.1071/MF10284)

[32] Mcdowall, R. M. 1968. Galaxias maculatus (jenyns), the New Zealand whitebait. Fisheries Research Division, Marine Department, Wellington, New Zealand.

[33] Chapman A, Morgan DL, Beatty SJ, Gill HS. 2006 Variation in life history of land-locked lacustrine and riverine populations of *Galaxias maculatus* (Jenyns 1842) in Western Australia. Environ. Biol. Fishes **77**, 21-37. (10.1007/s10641-006-9051-2)

[34] Stevens J, Hickford M, Schiel D. 2016 Evidence of iteroparity in the widely distributed diadromous fish inanga *Galaxias maculatus* and potential implications for reproductive output. J. Fish Biol. **89**, 1931-1946. (10.1111/jfb.13083) 27470074

[35] Allen GR, Midgley SH, Allen M. 2002 Field guide to the freshwater fishes of Australia. Western Australian Museum, Perth, Australia.

[36] Schneider CA, Rasband WS, Eliceiri KW. 2012 NIH Image to ImageJ: 25 years of image analysis. Nat. Methods **9**, 671-675. (10.1038/nmeth.2089) 22930834 PMC5554542

[37] Lenth R. 2023 emmeans: Estimated Marginal Means, aka Least-Squares Means. R package version 1.8.5. See https://CRAN.R-project.org/package=emmeans

[38] R Core Team A, Team RC. 2022. In R: A language and environment for statistical computing, pp. 2012. Vienna, Austria: R Foundation for Statistical Computing.

[39] Bates D, Mächler M, Bolker B, Walker S. 2014 Fitting linear mixed-effects models using lme4. arXiv preprint arXiv **1406**, 5823.

[40] Skeeles MR, Clark TD. 2023 Evidence for energy reallocation, not oxygen limitation, driving the deceleration in growth of adult fish. J. Exp. Biol. **226**, jeb246012. (10.1242/jeb.246012) 37334714

[41] Funk DH, Sweeney BW, Jackson JK. 2021 Oxygen limitation fails to explain upper chronic thermal limits and the temperature size rule in mayflies. J. Exp. Biol. **224**, jeb233338. 33288530 10.1242/jeb.233338

[42] Scheuffele H, Jutfelt F, Clark TD. 2021 Investigating the gill-oxygen limitation hypothesis in fishes: intraspecific scaling relationships of metabolic rate and gill surface area. Conserv. Physiol. **9**, coab040. (10.1093/conphys/coab040) 35692494 PMC8193116

[43] Audzijonyte A, Richards SA. 2018 The energetic cost of reproduction and its effect on optimal life-history strategies. Am. Nat. **192**, E150-E162. (10.1086/698655) 30205032

[44] Moffett ER, Fryxell DC, Benavente J, Kinnison M, Palkovacs E, Symons C, Simon K. 2022 The effect of pregnancy on metabolic scaling and population energy demand in the viviparous fish *Gambusia affinis*. Integr. Comp. Biol. **62**, 1419-1428. (10.1093/icb/icac099) 35767874

[45] Wu RS. 2009 Effects of hypoxia on fish reproduction and development. In Fish physiology *(eds JG Richards, AP Farrell, CJ Brauner), pp. 79–141. USA: Academic Press*.

[46] Servili A, Canario AV, Mouchel O, Muñoz-Cueto JA. 2020 Climate change impacts on fish reproduction are mediated at multiple levels of the brain-pituitary-gonad axis. Gen. Comp. Endocrinol. **291**, 113439. (10.1016/j.ygcen.2020.113439) 32061640

[47] Ineno T, Kodama R, Taguchi T, Yamada K. 2018 Growth and maturation in sterlet *Acipenser ruthenus* under high concentrations of dissolved oxygen. Fish. Sci. **84**, 605-612. (10.1007/s12562-018-1216-3)

[48] Texada MJ, Koyama T, Rewitz K. 2020 Regulation of body size and growth control. Genetics **216**, 269-313. (10.1534/genetics.120.303095) 33023929 PMC7536854

[49] Kolding J, Haug L, Stefansson S. 2008 Effect of ambient oxygen on growth and reproduction in Nile tilapia (*Oreochromis niloticus*). Can. J. Fish. Aquat. Sci. **65**, 1413-1424. (10.1139/F08-059)

[50] Nilsson GE, Dymowska A, Stecyk JA. 2012 New insights into the plasticity of gill structure. Respir. Physiol. Neurobiol. **184**, 214-222. (10.1016/j.resp.2012.07.012) 22846495

[51] Skeeles MR, Scheuffele H, Clark TD. 2023 Datasets and R scripts for: Supplemental oxygen does not improve growth but can enhance reproductive capacity of fish. Figshare. (10.6084/m9.figshare.21677174) PMC10618859 37909085

[52] Skeeles MR, Scheuffele H, Clark TD. 2023 Supplemental oxygen does not improve growth but can enhance reproductive capacity of fish. Figshare. (10.6084/m9.figshare.c.6879643) PMC10618859 37909085

